# Universal Filter Based on Compact CMOS Structure of VDDDA

**DOI:** 10.3390/s21051683

**Published:** 2021-03-01

**Authors:** Winai Jaikla, Fabian Khateb, Tomasz Kulej, Koson Pitaksuttayaprot

**Affiliations:** 1 Department of Engineering Education, Faculty of Industrial Education and Technology, King Mongkut’s Institute of Technology Ladkrabang, Bangkok 10520, Thailand; 2 Department of Microelectronics, Brno University of Technology, Technická 10, 601 90 Brno, Czech Republic; khateb@feec.vutbr.cz; 3 Faculty of Biomedical Engineering, Czech Technical University in Prague, nám.Sítná 3105, Kladno, 166 36 Prague, Czech Republic; 4 Department of Electrical Engineering, Technical University of Czestochowa, 42-201 Czestochowa, Poland; kulej@el.pcz.czest.pl; 5 Department of Technology Electronic, Faculty of Agricultural and Industrial Technology, Phetchabun Rajabhat University, Phetchabun 67000, Thailand; koson@pcru.ac.th

**Keywords:** VDDDA, biquad filter, operational transconductance amplifier, multiple-input technique

## Abstract

This paper proposes the simulated and experimental results of a universal filter using the voltage differencing differential difference amplifier (VDDDA). Unlike the previous complementary metal oxide semiconductor (CMOS) structures of VDDDA that is present in the literature, the present one is compact and simple, owing to the employment of the multiple-input metal oxide semiconductor (MOS) transistor technique. The presented filter employs two VDDDAs, one resistor and two grounded capacitors, and it offers low-pass: LP, band-pass: BP, band-reject: BR, high-pass: HP and all-pass: AP responses with a unity passband voltage gain. The proposed universal voltage mode filter has high input impedances and low output impedance. The natural frequency and bandwidth are orthogonally controlled by using separated transconductance without affecting the passband voltage gain. For a BP filter, the root mean square (RMS) of the equivalent output noise is 46 µV, and the third intermodulation distortion (IMD3) is −49.5 dB for an input signal with a peak-to peak of 600 mV, which results in a dynamic range (DR) of 73.2 dB. The filter was designed and simulated in the Cadence environment using a 0.18-µm CMOS process from Taiwan semiconductor manufacturing company (TSMC). In addition, the experimental results were obtained by using the available commercial components LM13700 and AD830. The simulation results are in agreement with the experimental one that confirmed the advantages of the filter.

## 1. Introduction

Analog filters are very useful in an analog signal processing system [1,2,3,4]. They are used to pass the desired frequency band from the input section to the output section. Therefore, the frequency filter is an essential element of any signal processing system that will be indispensable. Especially, in sensor applications, a filter is very important to detect the wanted signal, for example, in the phase sensitive detection [5], electrocardiographic (ECG) system [6], biosensors [7], etc. The biquad active filter has been a very popular research topic for last three decades. The main reason for the popularity of the biquad filter design is that it can be modified to obtain five filtering functions, which are the low-pass: LP, band-pass: BP, band-reject: BR, high-pass: HP and all-pass: AP functions. Compared to the first-order configuration, this filter can give only three LP, HP and BP functions, as well as the higher order filter, which mostly gives only one filtering response. The versatile biquad filter that gives multiple filtering functions within the same configuration is well-known as the universal or multifunction filter. In the open literature, this type of active filter has been proposed continuously [8,9,10,11,12].

The voltage differencing differential difference amplifier (VDDDA) [13] is a very useful and versatile building block for the voltage-mode active biquad filtering design. With the operational transconductance amplifier (OTA) at the input stage of the VDDDA, the filtering characteristics (for example, the passband voltage gain, bandwidth, quality factor, the cut-off frequency or phase response of the VDDDA-based filer) can be controlled via the transconductance (*g_m_*). Moreover, with the unity gain voltage differential difference amplifier (DDA) at the output stage of the VDDDA, it is very useful for the voltage-mode filter design without the use of additional or external voltage summing or a voltage differencing circuit, especially in the two-integrator loop filter design. Additionally, some VDDDA-based voltage-mode filters can cascade without the requirement of additional buffer devices. In this point of view, several active biquad filters using the VDDDA have been reported [13,14,15,16,17,18,19,20,21,22,23,24]. The voltage mode biquad filters using the voltage differential difference device (called the differential difference current conveyor transconductance amplifier (DDCCTA)) have been also proposed in references [25,26,27]. These biquad filters can be classified into two categories based on their design techniques, which are based on two integrator loops [13,14,15,17,19,20,21,22,25,27] and based on a passive resistor-inductor-capacitor (RLC) circuit [16,18,23,24,26]. The RLC based biquad filters proposed in [16,18,23,24] have very simple circuitry with a single VDDDA. However, these biquad filter realized from the RLC circuit have the following disadvantages: they consist of a floating capacitor [16,18,24,26], the natural frequency (*f*_0_) and quality factor (*Q*) are not orthogonally controlled by changing the transconductance of the VDDDA [16,18,23,24], high impedance nodes at the input stage and a low impedance node at the output stage are not obtained [16,18,23,24,26] and additional circuits such as the unity gain inverting voltage amplifier or double-gain voltage amplifier are required for obtaining many filtering functions [16,18,24,26]. Two-integrator loop-based biquad filters with high input impedance were obtained in references [13,14,15,17,19,20,21,22,27]. Additionally, a low output impedance property for all output nodes was obtained in references [19,21,22]. In [13,14,20,21,22], the *f*_0_ and *Q* were orthogonally controlled by the separated transconductance. However, the biquad filter proposed in references [13,14,20,21,22] requires three VDDDAs. Additionally, the passband voltage gain of three VDDDA-based filters in references [13,14,22] is not constant during tuning the *f*_0_ and *Q* for some filtering responses. The two-integrator loop filter in references [15,17] cannot provide five filter responses. Additionally, two VDDDA-based biquad filters in references [15,17,19] and the biquad filter using DDCCTA in references [25,27] cannot achieve the orthogonal control of the *f*_0_ and *Q* by separated transconductance. As reviewed above, it was found that the two VDDDA-based universal biquad filters with the feature of orthogonal control of the *f*_0_ and *Q* by the separated transconductance were been available in the open literature.

The multiple-input MOS transistor (MI-MOST) is an effective technique that may reduce the count of the transistors needed to build some active blocks and simplify their CMOS topologies. This technique was, for the first time, presented and experimentally confirmed by Khateb et al. [28,29,30]. Recently, several active functional blocks, such as the differential difference amplifier (DDA) [28], differential difference current conveyors (DDCC) [30,31,32], fully differential difference amplifier (FDDA) [33] and multiple-input OTA [6,34,35,36], were designed based on this technique. It was shown that applications based on the MI-MOST technique can be realized with less numbers of active blocks, smaller chip areas and reduced power consumptions compared to conventional designs [6,28,29,30,31,32,33,34,35,36].

In the past, a multiple-input OTA was used to reduce the number of components, silicon area, and power dissipation in the OTA-C filter design where a third-order elliptic low-pass filter was built by five double-input OTAs instead of seven single-input OTAs [37]. Another example is the use of a two-input-stage OTA instead of a conventional OTA to build a leapfrog realization of a seventh-order elliptic filter in order to facilitate the signal addition required in the feedback paths of the leapfrog realization [38]. In both the aforementioned examples, additional active transistors were used to realize the multiple-input terminals. Another way to obtain multiple-input MOST is the utilizing of a multiple-input floating-gate transistor (MIFG) [39]. However, this technique is based on charge conservation; hence, it cannot be used in CMOS technologies with gate leakage [40].

**Figure 1:**
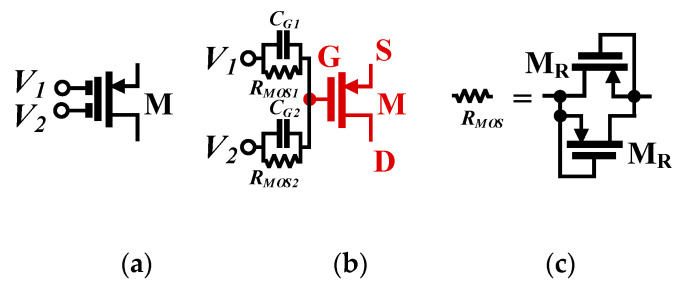
The multiple-input metal oxide semiconductor transistor (MOST): (**a**) symbol, (**b**) realization and (**c**) realization of *R_MOS_* [28].

Figure 1 shows an example of multiple-input gate-driven MOST with two inputs. Each input (*V*_1_ and *V*_2_) is connected to the gate (G) of the MOST (M) by a parallel connection of an input capacitor (*C*_1_ and *C*_2_) and high resistance (*R*_*MOS*1_ and *R*_*MOS*2_) created by two MOST (M_R_) operating as a diode in the cut-off region. Due to using the transistor M_R_, the chip area is saved. Note that the multiple-input technique could be applied to the gate terminal [6,36], bulk terminal [28], bulk-gate terminal [34] or bulk-quasi-floating-gate terminal of the MOST [29]. However, unlike the aforementioned multiple-input techniques, the multiple-input gate-driven MOST offers the advantage of that regardless of the CMOS technology used; both transistor types (N-MOS and PMOS) could be created with a multiple-input signal.

This paper presents a universal filter based on the compact CMOS structure of the multiple-input gate-driven VDDDA. The paper is organized as follows: Section 2 presents the CMOS structure of the VDDDA and the proposed universal filter. Section 3 and Section 4 present the simulation and experimental results, respectively. The comparison of the proposed biquad filter with other filters using VDDDA is discussed and explained in Section 5, and finally, Section 6 concludes the paper.

## 2. Proposed Universal Filter

### 2.1. Basic Concept of the VDDDA

**Figure 2:**
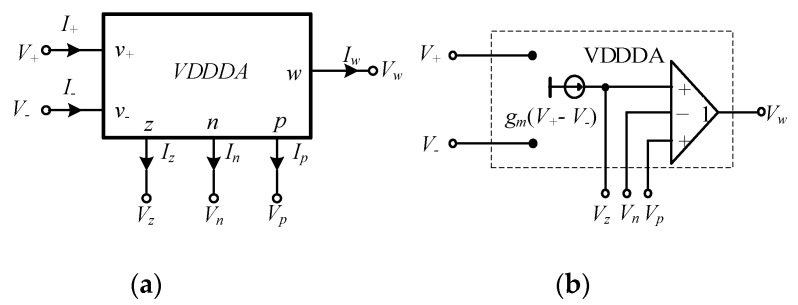
Voltage differencing differential difference amplifier (VDDDA): (**a**) electrical symbol and (**b**) equivalent circuit [13].

The VDDDA is a connection of OTA and a unity gain DDA. The basic version of the VDDDA with circuit symbols, as in Figure 2a, is an analog functional block with five terminals. The high-impedance input voltage terminals are *v_+_*, *v_−_*, *z*, *n* and *p*. The low-impedance output voltage terminal is *w*. Note that the *z* terminal is also the output current terminal. An ideal corresponding equivalent circuit of the VDDDA is illustrated in Figure 2b. The circuit performance is described by the matrix Equation (1):

(1) $$I_{+}=I_{-}=I_{n}=I_{p}=0;\;I_{z}=g_{m}\left(V_{+}-V_{-}\right);\;V_{w}=V_{z}-V_{n}+V_{p},$$

### 2.2. The CMOS Structure of the VDDDA

**Figure 3:**
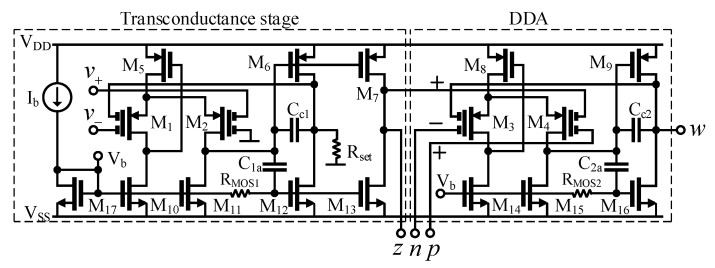
The CMOS structure of the VDDDA.

The VDDDA consists of a transconductance stage (*g_m_*) followed by a differential difference amplifier (DDA), as shown in Figure 3. The transconductance stage is formed by a differential stage (M_1_, M_2_, M_5_, M_10_ and M_11_) and a doubled output stage (M_6_, M_12_, M_7_ and M_13_). Thanks to the negative feedback between the output (drain of M_6_) and the input terminals of M_1_, the linearity of the transconductance is increased. The capacitor *C*_1*a*_ and the MOS resistor *R*_*MOS*1_ are used to obtain a class AB output stage. The capacitor *C*_*c*1_ creates the compensation network of the transconductor. The resistor *R_set_* is used to set the transconductance value in such a way that *g_m,set_* ≈ 1/*R_set_* for *R_set_* >> 1/*g_m_*, where *g_m_* is the internal transconductance of this stage [41]. Note that the tuning mechanism is very simple; the difference of the input voltages (*V_+_* − *V_−_*) will appear across *R_set_* due to the negative unity feedback. The resistor current *I_Rset_* will be controlled by the value of *R_set_*. The output current *I_z_* is a copy of *I_Rset_*. The DDA is created in similar manner as the transconductance stage. The bias current *I_b_* and the transistor M_17_ create the bias current and voltage needed for the circuit. It is worth noting that the minimum voltage supply of the proposed VDDDA structure is given by one gate-source and one drain-source voltage, i.e., V_DDmin_ = V_GSM5_ + V_DSM10_. Hence, the structure is suitable for low-voltage supply applications.

### 2.3. The Universal Biquad Filter based on VDDDAs

**Figure 4:**
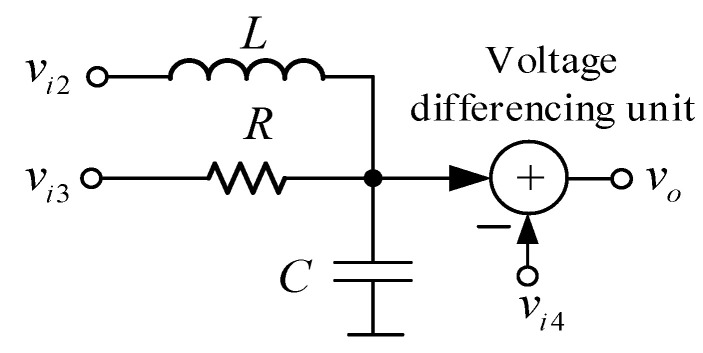
Method to design the proposed biquad filter.

Most of the published papers on universal biquad filters have not shown the method to design the topology. Therefore, new researchers or designers do not understand how to get the completed circuits. In this paper, a simple method to design the biquad filter is also presented to achieve the important goal of this design, which is to use only two VDDDAs to get the orthogonal tune of the quality factor and the natural frequency by using separated transconductance. The proposed biquad universal filter is designed from the parallel RLC circuit connecting with the voltage differencing circuit, as shown in Figure 4. There are three input voltages applied to the circuit: *v*_*i*2_ is applied through the inductor, *v*_*i*3_ is applied to the resistor and *v*_*i*4_ is applied at the inverting input of the voltage differencing circuit. For a conventional design, other input voltages can be applied through the capacitor. However, using a grounded capacitor is required for this design to ensure the reduction in fabrication space and the compensation of parasitic effects.

Considering the circuit shown in Figure 4, the following output voltage, *v_o_*, is obtained:

(2) $$v_{o}=\frac{\frac{1}{LC}v_{i2}+s\frac{1}{CR}v_{i3}-\left(s^{2}+s\frac{1}{CR}+\frac{1}{LC}\right)v_{i4}}{s^{2}+s\frac{1}{CR}+\frac{1}{LC}},$$

Based on Equation (2), several filter responses can be obtained from the same topology by appropriately applying the input signal to the input voltage nodes *v*_*i*2_, *v*_*i*3_ and *v*_*i*4_ of the filter, and this detail will be described later. The natural frequency, bandwidth (*BW*) and quality factor for Equation (2) are obtained by

(3) $$\omega_{0}=\sqrt{\frac{1}{LC}},$$

(4) $$BW=\frac{1}{RC},$$

and

(5) $$Q=R\sqrt{\frac{C}{L}},$$

Equations (3) and (4) indicate that the *ω*_0_ and *BW* (or the *Q*) are orthogonally controlled. It is vital to note that the *Q* is controlled through the resistor *R* without affecting the natural frequency. To get the required natural frequency and bandwidth for the practical design, the natural frequency must be first designed by setting the inductance (*L*) and capacitance (*C*) values. Then, the required bandwidth or quality factor can be achieved by independently setting the resistance value without disturbing the natural frequency.

**Figure 5:**
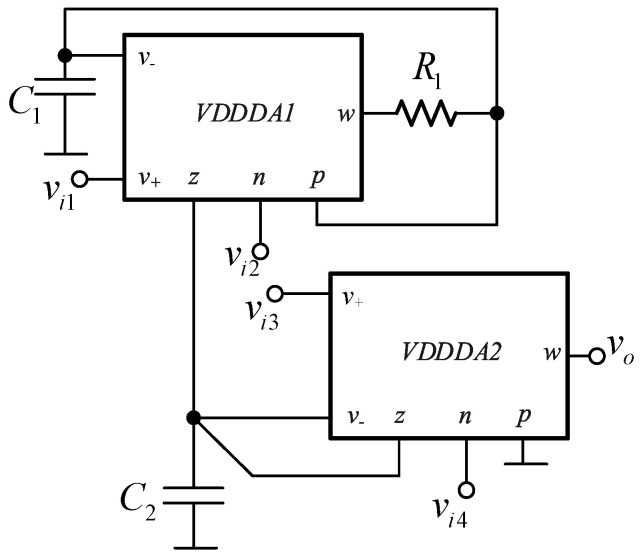
The universal filter based on the VDDDA.

Considering the method to design the filter in Figure 4, there are four sub-circuits, which are the inductor, resistor, grounded capacitor and the voltage summing circuit. These passive inductor and resistors can be replaced by active simulators using the VDDDA. The active inductor used in this design is modified from the floating lossless inductance simulator using two VDDDAs proposed in reference [42]. Only one VDDDA is required for the active inductor in our work. Additionally, the active resistor and voltage differencing circuit can be realized from one VDDDA. Based on this principle, the proposed universal biquad filter is illustrated in Figure 5. The proposed filter comprises two VDDDAs, two grounded capacitors and one resistor, where the VDDDA_1_, *C*_1_ and *R*_1_ operate as the active inductors. The active resistor and voltage differencing circuit is constructed from VDDDA_2_, and the grounded capacitor *C*_2_ acts as *C* in Figure 4. There are four input voltage nodes: *v*_*i*1_, *v*_*i*2_, *v*_*i*3_ and *v*_*i*4_, which is slightly different from the principle in Figure 4. The input voltage nodes *v*_*i*2_, *v*_*i*3_ and *v*_*i*4_ are the same as the principle in Figure 4. The additional *v*_*i*1_ node is added to get the all-pass response, which will be given more detail later. It is found that all input voltage nodes of the proposed universal biquad filter are high impedance, while the output voltage (*v_o_*) node is low impedance. The proposed universal biquad filter can cascade without the requirement of any voltage buffers at the input and output stages. Moreover, it consists of all the grounded capacitors, which ensures a reduction in the fabrication space and compensation of the parasitic effects.

Considering the circuit shown in Figure 5, the following output voltage, *v_o_*, is obtained.

(6) $$v_{o}=\frac{s\frac{g_{m1}}{C_{2}}v_{i1}+\frac{g_{m1}}{C_{1}C_{2}R_{1}}v_{i2}+s\frac{g_{m2}}{C_{2}}v_{i3}-\left(s^{2}+s\frac{g_{m2}}{C_{2}}+\frac{g_{m1}}{C_{1}C_{2}R_{1}}\right)v_{i4}}{s^{2}+s\frac{g_{m2}}{C_{2}}+\frac{g_{m1}}{C_{1}C_{2}R_{1}}},$$

Based on Equation (6), several filter responses can be given from the same filtering topology in Figure 5 by applying the appropriate input voltage signal to the input nodes of the filter as follows:

- Noninverting low-pass filter with unity voltage gain is given at the output voltage node *v_o_* of the proposed filter by applying the input signal into the input voltage node *v*_*i*2_ while the other input voltage nodes are grounded.
- Noninverting band-pass filter with unity voltage gain is given at the output voltage node *v_o_* of the proposed filter by applying the input signal into the input voltage node *v*_*i*3_ while the other input voltage nodes are grounded.
- Inverted high-pass filter with unity voltage gain is given at the output voltage node *v_o_* of the proposed filter by applying the input signal into the input voltage nodes *v*_*i*2_, *v*_*i*3_ and *v*_*i*4_ while the input voltage node *v*_*i*1_ is grounded.
- Inverted band-stop filter with unity voltage gain is given at the output voltage node *v_o_* of the proposed filter by applying the input signal into the input voltage nodes *v*_*i*3_ and *v*_*i*4_ while other nodes are grounded.
- Inverted all-pass filter with unity voltage gain is given at the output voltage node *v_o_* of the proposed filter by setting *g*_*m*1_ = *g*_*m*2_ and applying the input signal into the input voltage nodes *v*_*i*1_, *v*_*i*3_ and *v*_*i*4_ while the input voltage node *v*_*i*2_ is grounded. Although it requires the matching conditions of *g*_*m*1_ and *g*_*m*2_, this is the active matching condition that is easier to control than the passive matching one.
- Inverted all-pass filter without the matching condition is given at the output voltage node *v_o_* of the proposed filter by connecting the *z* terminal to the *p* terminal of the VDDDA_2_, then applying the input signal into the input volage nodes *v*_*i*3_ and *v*_*i*4_ while the other input voltage nodes are grounded.

From the above statement, it is found that the selection of filter responses does not require additional circuits—for example, inverting or double-gain amplifiers. The natural frequency, bandwidth and quality factor for Equation (6) are obtained by

(7) $$\omega_{0}=\sqrt{\frac{g_{m1}}{C_{1}C_{2}R_{1}}},$$

(8) $$BW=\frac{g_{m2}}{C_{2}},$$

and

(9) $$Q=\frac{1}{g_{m2}}\sqrt{\frac{g_{m1}C_{2}}{C_{1}R_{1}}},$$

Equations (7) and (8) indicate that the *ω*_0_ and *BW* (or the *Q* in Equation (9)) are orthogonally controlled. In a practical design to get the required natural frequency and bandwidth, the natural frequency must be first designed by setting the *g*_*m*1_, *R*_1_, *C*_1_ and *C*_2_. Then, the required bandwidth or quality factor can be achieved by independently setting the *g_m_*_2_ without disturbing the natural frequency.

### 2.4. Effects of Nonideal VDDDA Characteristics

The nonideal properties of the VDDDA will affect the performances of the proposed universal biquad filter. Therefore, these nonideal cases will be considered and studied in this section. There are two nonideal characteristics, which are the voltage tracking errors and parasitic impedances, at the VDDDTA terminals. Firstly, the voltage tracking error in the voltage differencing stage of the VDDDA will be considered. The VDDDA property with the voltage tracking errors is given by

(10) $$\left(\begin{array}{c} I_{+}\\ I_{-}\\ I_{z}\\ I_{n}\\ I_{p}\\ V_{w} \end{array}\right)=\left(\begin{array}{cccccc} 0 & 0 & 0 & 0 & 0 & 0\\ 0 & 0 & 0 & 0 & 0 & 0\\ g_{m} & g_{m} & 0 & 0 & 0 & 0\\ 0 & 0 & 0 & 0 & 0 & 0\\ 0 & 0 & 0 & 0 & 0 & 0\\ 0 & 0 & \beta_{z} & -\beta_{n} & \beta_{p} & 0 \end{array}\right)\left(\begin{array}{c} V_{v+}\\ V_{v-}\\ V_{z}\\ V_{n}\\ V_{p}\\ I_{w} \end{array}\right),$$

Here, *β_z_* = 1 − *ε_z_* and *ε_z_* (|*ε_z_*| <<1) denotes the voltage tracking error from the *z* to *w* terminal, *β_n_* = 1 − *ε_n_* and *ε_n_* (|*ε_n_*| << 1) denotes the voltage tracking error from the *n* to *w* terminal and *β_p_* = 1 − *ε_p_* and *ε_p_* (|*ε_n_*| << 1) denotes the voltage tracking error from the *p* to *w* terminal. Considering these errors, the output voltage of the proposed universal filter can be expressed as follows:

(11) $$v_{o}=\frac{\left[\begin{array}{l} \left(1-\varepsilon_{z2}\right)\left(s\frac{g_{m1}}{C_{2}}+\frac{\varepsilon_{p1}g_{m1}}{C_{1}C_{2}R_{1}}\right)v_{i1}+\left(1-\varepsilon_{n1}\right)\left(1-\varepsilon_{z2}\right)\frac{g_{m1}}{C_{1}C_{2}R_{1}}v_{in2}+\\ \left(1-\varepsilon_{z2}\right)\left(s\frac{g_{m2}}{C_{2}}+\frac{\varepsilon_{p1}g_{m2}}{C_{1}C_{2}R_{1}}\right)v_{i3}-\left(1-\varepsilon_{n2}\right)D^{*}(s)v_{i4} \end{array}\right]}{D^{*}(s)},$$

where

(12) $$D^{*}(s)=s^{2}+s\left[\frac{\varepsilon_{p1}}{C_{1}R_{1}}+\frac{1}{C_{2}}g_{m2}\right]+\frac{\varepsilon_{p1}g_{m2}+\left(1-\varepsilon_{z1}\right)g_{m1}}{C_{1}C_{2}R_{1}},$$

Therefore, the *ω*_0_, *BW* and *Q* with nonideal gains are, respectively, given as

(13) $$\omega_{0}^{*}=\sqrt{\frac{\varepsilon_{p1}g_{m2}+\left(1-\varepsilon_{z1}\right)g_{m1}}{C_{1}C_{2}R_{1}}},$$

(14) $$BW^{*}=\frac{\varepsilon_{p1}}{C_{1}R_{1}}+\frac{1}{C_{2}}g_{m2},$$

and

(15) $$Q^{*}=\frac{C_{1}C_{2}R_{1}}{\varepsilon_{p1}C_{2}+C_{1}R_{1}g_{m2}}\sqrt{\frac{\varepsilon_{p1}g_{m2}+\left(1-\varepsilon_{z1}\right)g_{m1}}{C_{1}C_{2}R_{1}}}.$$

It is noticeable that the voltage gain, natural frequency, bandwidth and the quality factor are affected by the voltage tracking errors. Additionally, it is found in Equation (14) that the transconductance, *g*_*m*2_ will slightly affect the natural frequency due to the *ε*_*p*1_. However, these voltage tracking errors are much less than one; their effects can be neglected at low-frequency operations.

**Figure 6:**
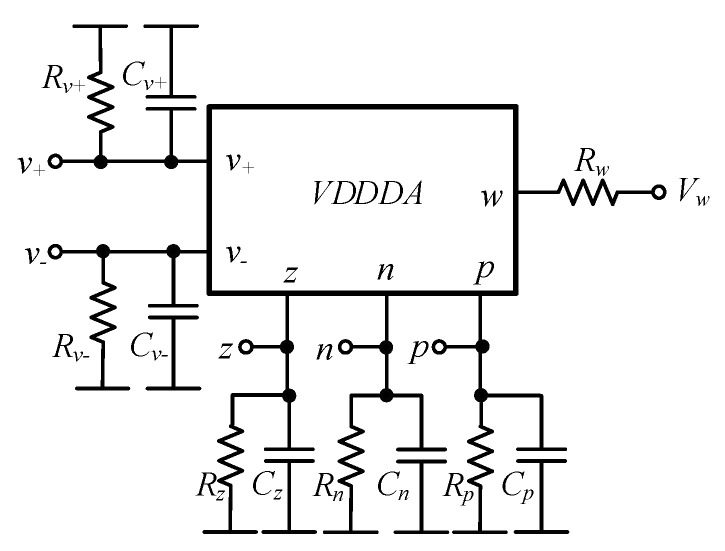
The parasitic effects on the VDDDA.

The effects of parasitic impedances at the input and output terminals of the VDDDA are considered next. These parasitic impedances are the parallel *C_+_* and *R_+_* at the *v_+_* terminal, the parallel *C-* and *R−* at the *v_−_* terminal, the parallel *C_z_* and *R_z_* at the *z* terminal, the parallel *C_n_* and *R_n_* at the *n* terminal, the parallel *C_p_* and *R_p_* at the *p* terminal and the *R_w_* (series at the *w* terminal) at the low output impedance *w* terminal, as shown in Figure 6.

Considering theses parasitic impedances, the output voltage under the parasitic effect is given by

(16) $$v_{o}=\frac{\left(s\frac{g_{m1}}{C_{2}^{*}R_{1}^{*}}+\frac{G_{1}^{*}g_{m1}}{C_{1}^{*}C_{2}^{*}R_{1}^{*}}\right)v_{i1}+g_{m1}v_{in2}+\left(s\frac{g_{m2}}{C_{2}^{*}R_{1}^{*}}+\frac{G_{1}^{*}g_{m2}}{C_{1}^{*}C_{2}^{*}R_{1}^{*}}\right)v_{i3}-D^{**}(s)v_{n2}}{D^{**}(s)},$$

where

(17) $$D^{**}(s)=s^{2}+s\left(\frac{G_{1}^{*}}{C_{1}^{*}}+\frac{G_{2}^{*}}{C_{2}^{*}}+\frac{g_{m2}}{C_{2}^{*}}\right)+\frac{G_{1}^{*}g_{m2}R_{1}+G_{1}^{*}G_{2}^{*}R_{1}^{*}+g_{m1}}{C_{1}^{*}C_{2}^{*}R_{1}^{*}},$$

and $C_{1}^{*}=C_{1}+C_{-1}+C_{p1}$, $C_{2}^{*}=C_{2}+C_{z1}+C_{-2}+C_{z2}$, $G_{1}^{*}=G_{-1}+G_{p1}$, $G_{2}^{*}=G_{z1}+G_{-2}+G_{z2}$, $R_{1}^{*}=R_{1}+R_{w1}$, *G*_*−*1_ = 1/*R*_*−*1_, *G*_*p*1_ = 1/*R*_*p*1_, *G*_*z*1_ = 1/*R*_*z*1_, *G*_*−*2_ = 1/*R*_*−*2_ and *G*_*z*2_ = 1*/R*_*z*2_. The *ω*_0_, BW and *Q* with parasitic effects are respectively given as

(18) $$\omega_{0}^{**}=\sqrt{\frac{G_{1}^{*}g_{m2}R_{1}+G_{1}^{*}G_{2}^{*}R_{1}^{*}+g_{m1}}{C_{1}^{*}C_{2}^{*}R_{1}^{*}}},$$

(19) $$BW^{**}=\frac{G_{1}^{*}}{C_{1}^{*}}+\frac{G_{2}^{*}}{C_{2}^{*}}+\frac{g_{m2}}{C_{2}^{*}},$$

and

(20) $$Q^{**}=\frac{C_{1}^{*}C_{2}^{*}R_{1}^{*}}{C_{2}^{*}G_{1}^{*}R_{1}+C_{1}^{*}G_{2}^{*}R_{1}^{*}+C_{1}^{*}g_{m2}R_{1}^{*}}\sqrt{\frac{G_{1}^{*}g_{m2}R_{1}+G_{1}^{*}G_{2}^{*}R_{1}^{*}+g_{m1}}{C_{1}^{*}C_{2}^{*}R_{1}^{*}}}.$$

It is noticeable that the voltage gain, natural frequency, bandwidth, the quality factor and operational frequency range of the proposed filter are affected by the parasitic impedances in the VDDDA. Additionally, it is found from Equation (20) that the transconductance, *g*_*m*2_, will slightly affect the natural frequency due to the parasitic impedance, $G_{1}^{*}$. However, the effect of the parasitic capacitances can be minimized by setting the value of *C*_1_ >> *C*_*−*1_ and *C*_*p*1_ and *C*_2_ >> *C*_*z*1_, *C*_*−*2_ and *C*_*z*2_, while the parasitic resistance *R*_*w*1_ can be minimized by setting the value of *R*_1_ >>*R*_*w*1_. As mentioned above, using grounded capacitors is advantageous for the compensation of parasitic effects.

## 3. Simulation Results

The CMOS circuit of the VDDDA and the filter application were designed and simulated in the Cadence environment using a 0.18-µm CMOS process from TSMC. The voltage supply is *V_DD_* = −*V_SS_* = 0.9 V, the bias current is *I_b_* = 50 µA and the total power consumption is 0.99 mW. The transistors aspect ratio of the VDDDA shown in Figure 3 are M_1_ − M_4_ = 90 µm/3 µm; M_5_ − M_9_ = 2 × 90 µm/3 µm; M_10_, M_11_, M_14_, M_15_ and M_17_ = 30 µm/3 µm; M_12_, M_13_ and M_16_ = 2 × 30 µm/3 µm and M_R_ = 4 µm/5 µm. The values of the passive components are *C*_*c*1_, *C*_*c*2_, *C*_1*a*_ and *C*_2*a*_ = 2.6 pF and *C_G_* = 0.5 pF.

**Figure 7:**
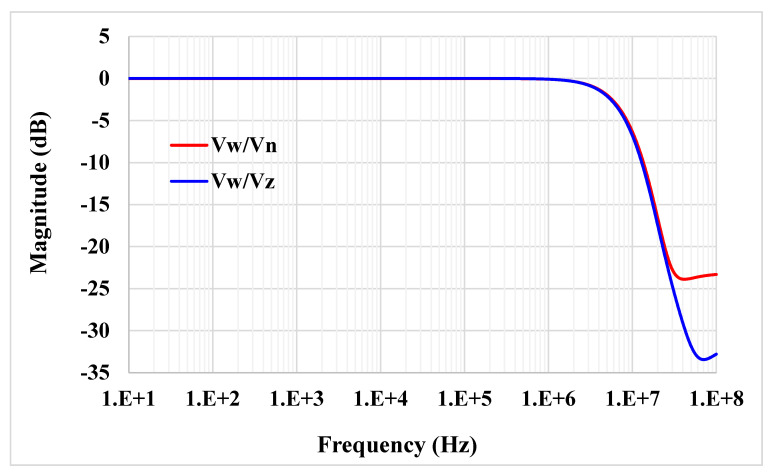
The frequency responses of |*V_w_*/*V_n_*| and |*V_w_*/*V_z_*|.

**Figure 8:**
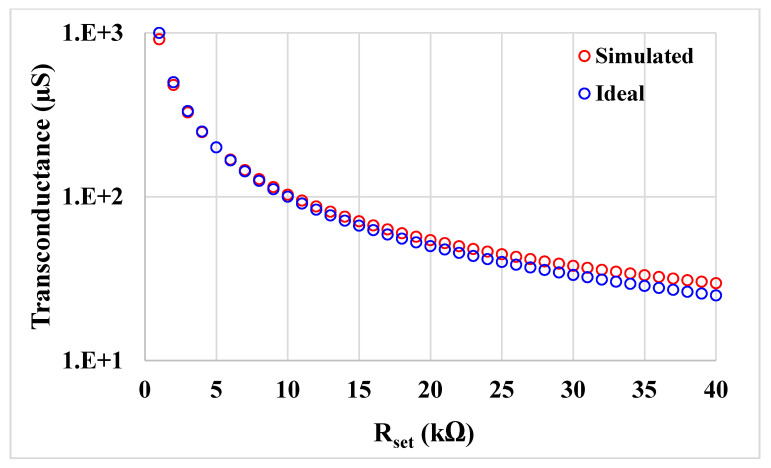
The transconductance value of the simulated and ideal transconductor versus *R_set._*

Figure 7 shows the frequency responses of *V_w_*/*V_n_* and *V_w_*/*V_z_* (*V_w_*/*V_p_*), where the gain at low frequency is −7 mdB and 5 mdB and the −3 dB bandwidth is 6.3 MHz and 6.1 MHz, respectively. Figure 8 shows the relation of the simulated and ideal transconductance versus the *R_set_*. Note that the curves match for the lower value of *R_set_*.

**Figure 9:**
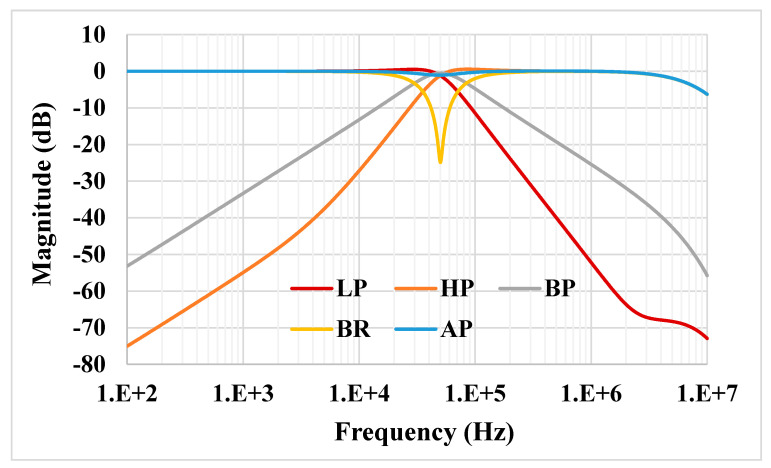
Frequency characteristics of the proposed universal filter.

**Figure 10:**
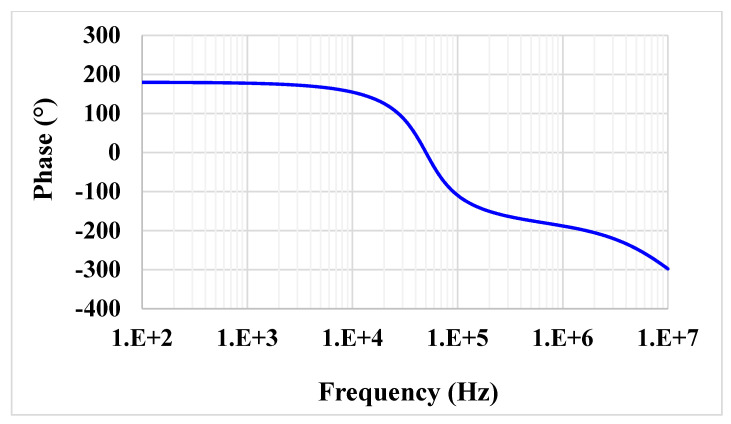
Phase characteristics of the all-pass (AP) filter.

**Figure 11:**
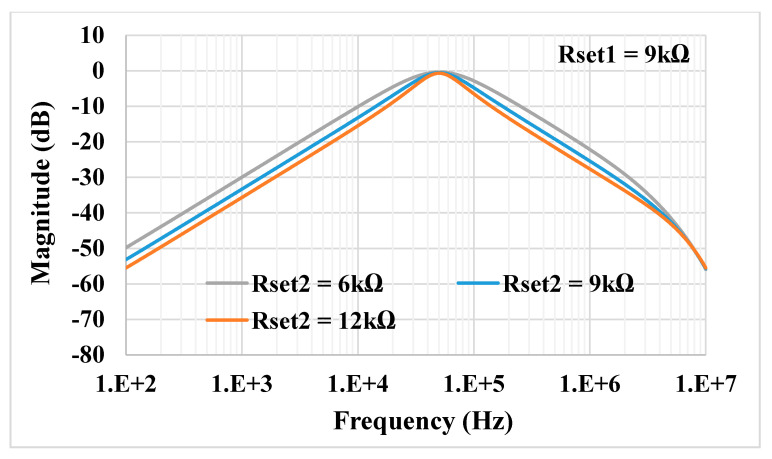
Tunability of the band-pass (BP) filter for different *R*_*set*2_ and the constant *R*_*set*1_.

**Figure 12:**
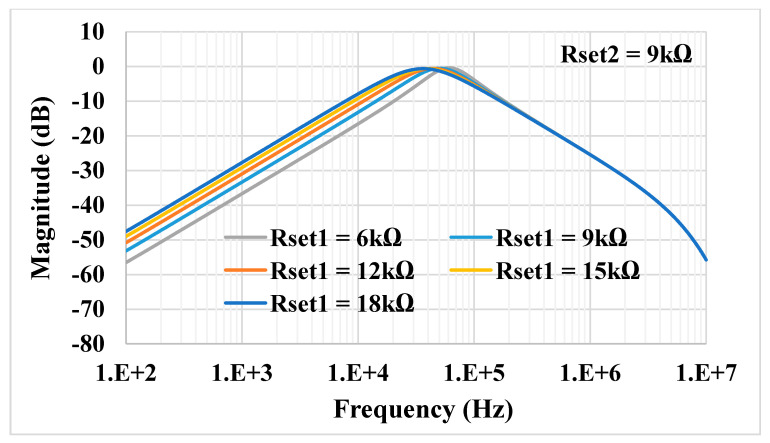
Tunability of the BP filter for different *R*_*set*1_ and the constant *R*_*set*2_.

**Figure 13:**
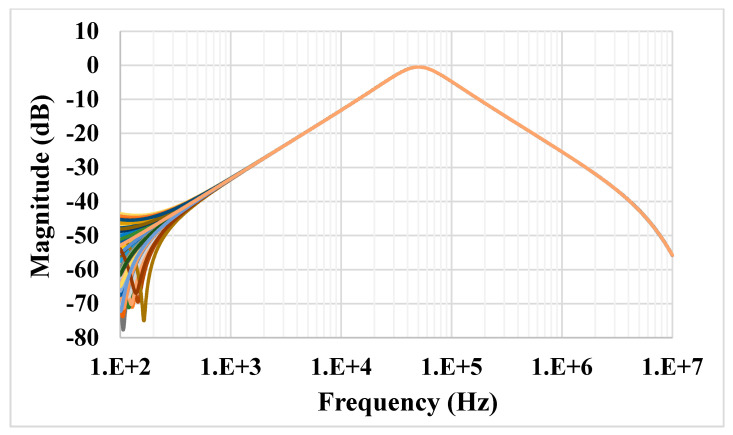
Monte Carlo analysis of the frequency characteristics of the BP filter with 200 runs.

**Figure 14:**
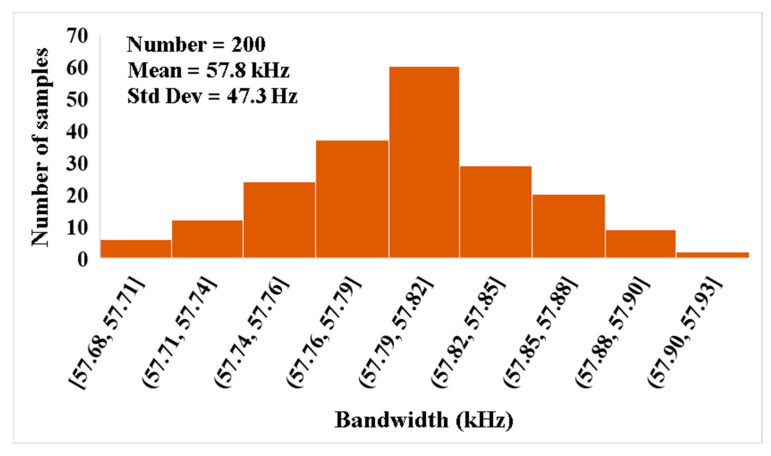
The histogram of the bandwidth of the BP filter with 200 runs of the Monte Carlo analysis.

For the proposed filter in Figure 5, the values of the passive components were selected as *C*_1_ = *C*_2_ = 335 pF, *R*_1_ = 10 kΩ, and the value of the resistor of the transconductors was *R*_*set*1_ = *R*_*set*2_ = 9 kΩ. Figure 9 shows the frequency characteristics of the LP, HP, BP, BR and AP filters, while the phase response of the AP filter is shown in Figure 10. However, for the BR response, the attenuation was obtained around −26 dB due to nonideal characteristics of the VDDDA, as explained in Section 2.4. The simulated natural frequency was *f*_0_ = 50 kHz. Figure 11 shows the tunability of the BP filter for *R*_*set*2_ = 6 kΩ, 9 kΩ and 12 kΩ, while *R*_*set*1_ = 9 kΩ. Figure 12 shows the tunability of the BP filter for *R*_*set*1_ = 6 kΩ, 9 kΩ, 12 kΩ, 15 kΩ and 18 kΩ, while *R*_*set*2_ = 9 kΩ. The frequency *f*_0_ was 60.9 kHz, 50.1 kHz, 44.1 kHz, 39.3 kHz and 36.3 kHz, respectively. The Monte Carlo analysis (including transistors and passive device mismatches) of the BP filter with 200 runs is shown in Figure 13. The curves are overlapped and confirm the stability of the circuit. Figure 14 shows the histogram of the bandwidth (BW) of the BP filter with 200 runs of the Monte Carlo analysis. While the mean value is 57.8 kHz, the standard deviation is only 47.3 Hz.

**Figure 15:**
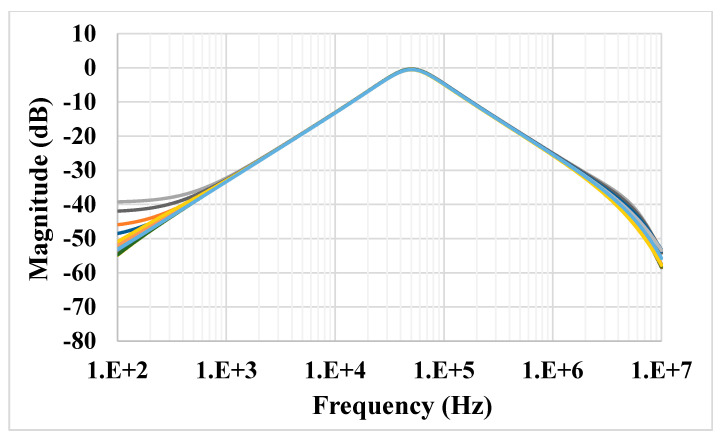
The process, voltage and temperature (PVT) corner analysis of the BP filter.

**Figure 16:**
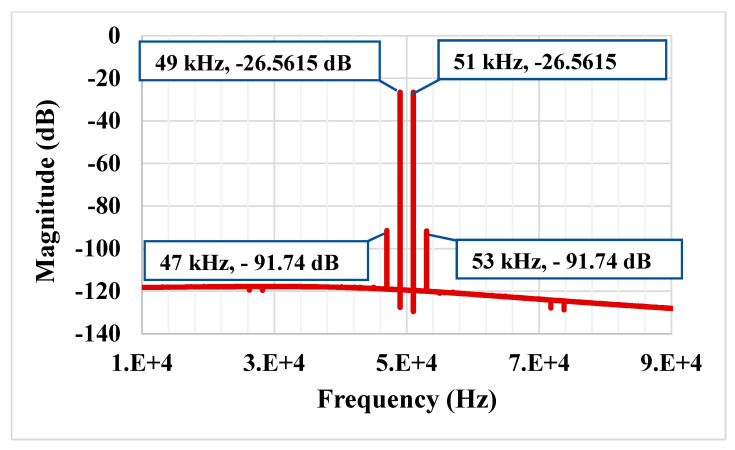
The spectrum of the output signal of the BP filter.

**Figure 17:**
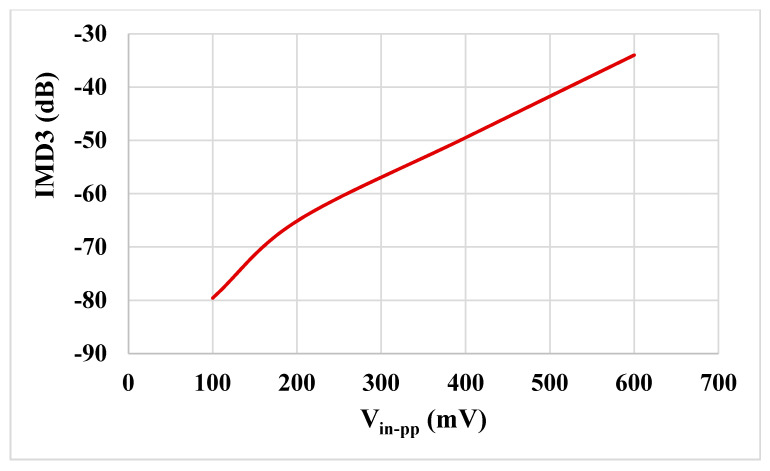
The third intermodulation distortion (IMD3) of the BP filter versus the peak-to-peak value of the input signal.

**Figure 18:**
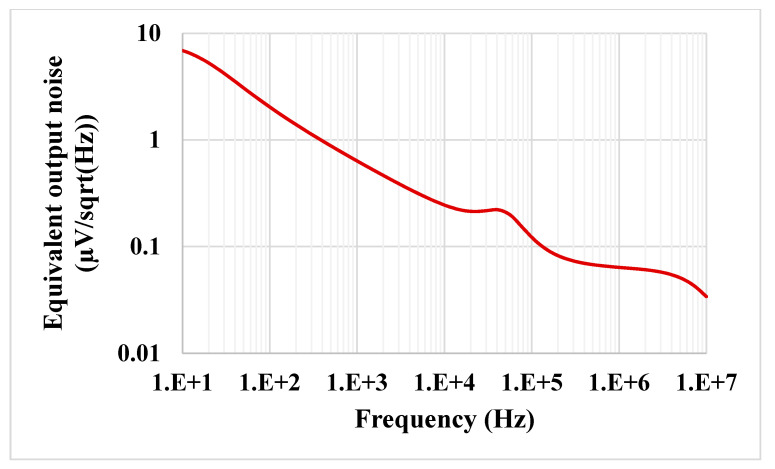
The equivalent output noise of the BP filter.

The results of the process, voltage and temperature (PVT) corner analysis of the BP filter are shown in Figure 15. The process corners for the MOST were fast-fast, fast-slow, slow-fast and slow-slow, for capacitor and resistor corners were fast and slow, the voltage supply corners VDD = −VSS were 890 mV and 910 mV and, finally, the temperature corners were −20 and 80 °C. While the nominal value of the BW is 57.8 kHz, the minimum BW is 57.17 kHz, and the maximum BW is 58.55 kHz under all corner variations. To test the third intermodulation distortion of the BP filter, two closely spaced tones were applied to the input of the BP filter. The first tone was a sine wave signal with 100-mVpp @ 49 kHz and the second tone with 100-mVpp @ 51 kHz. The spectrum of the output signal is shown in Figure 16. The third intermodulation distortion (IMD3) was −65.148 dB. Figure 17 shows the IMD3 of the BP filter versus the peak-to-peak value of the input signal. It is clear that the filter offers IMD3 of −34 dB for the 600-mVpp input signal. As shown in Figure 18, the RMS value of the output noise integrated in the pass band of the filter (29.11 kHz–86.9 kHz) is 46 µVrms, and the RMS value of the input signal for 2% IMD3 is 0.2121 V; hence, the dynamic range of the BP filter is 73.27 dB.

## 4. Experimental Results

**Figure 19:**
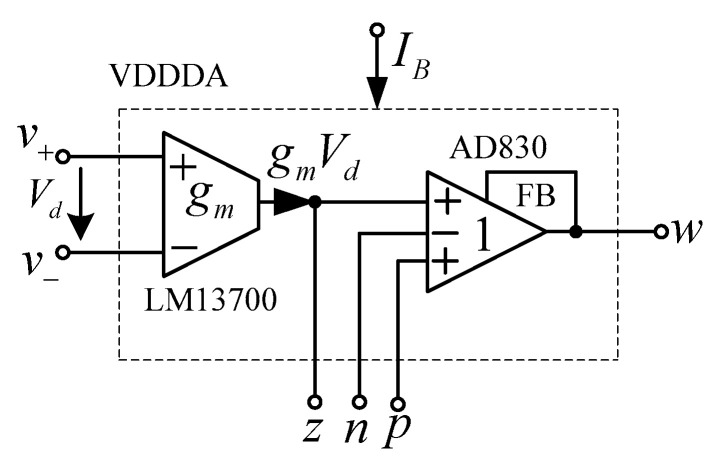
VDDDA based on the commercial components.

**Figure 20:**
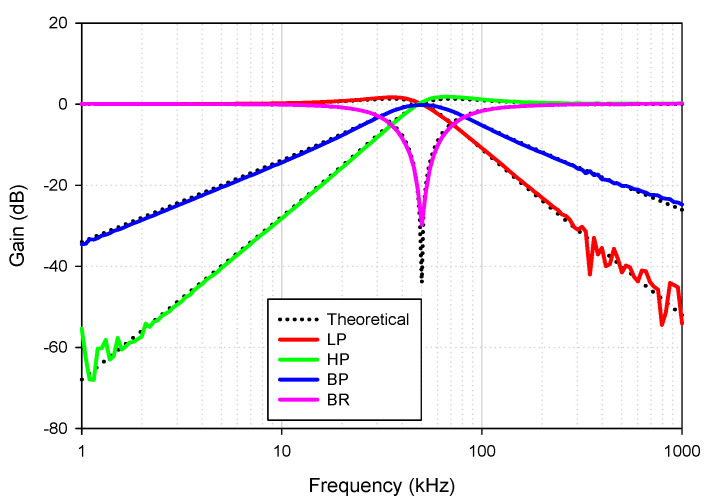
Experimental frequency characteristics of the proposed universal filter.

**Figure 21:**
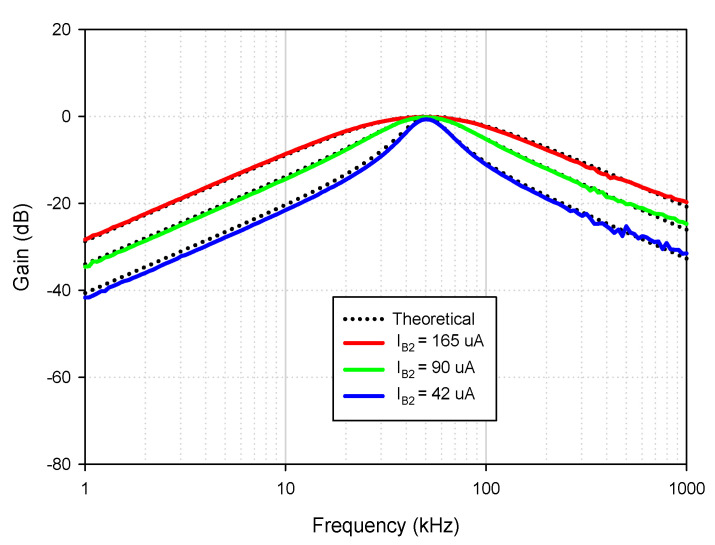
Experimental BP response for different bias current (I_B2_) values (*I*_*B*1_ = 155 μA, *R*_1_ = 1 kΩ and *C*_1_ = *C*_2_ = 5.5 nF).

**Figure 22:**
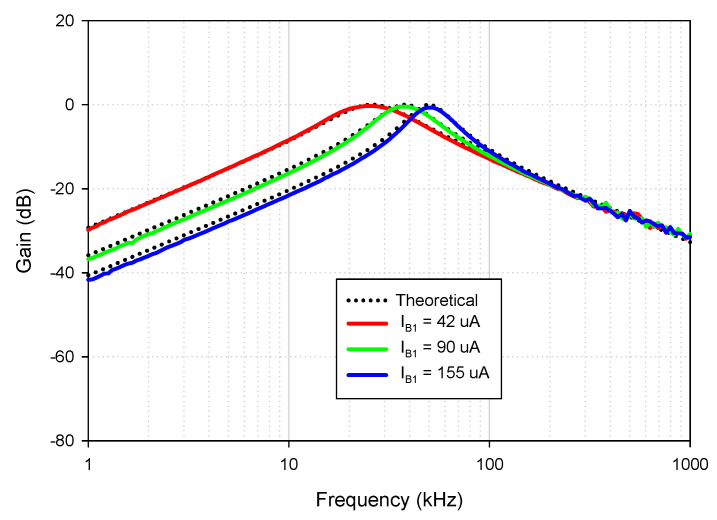
Experimental BP responses for different I_B1_ values (*I*_*B*2_ = 195 μA, *R*_1_ = 1 kΩ and *C*_1_ = *C*_2_ = 5.5 nF).

**Figure 23:**
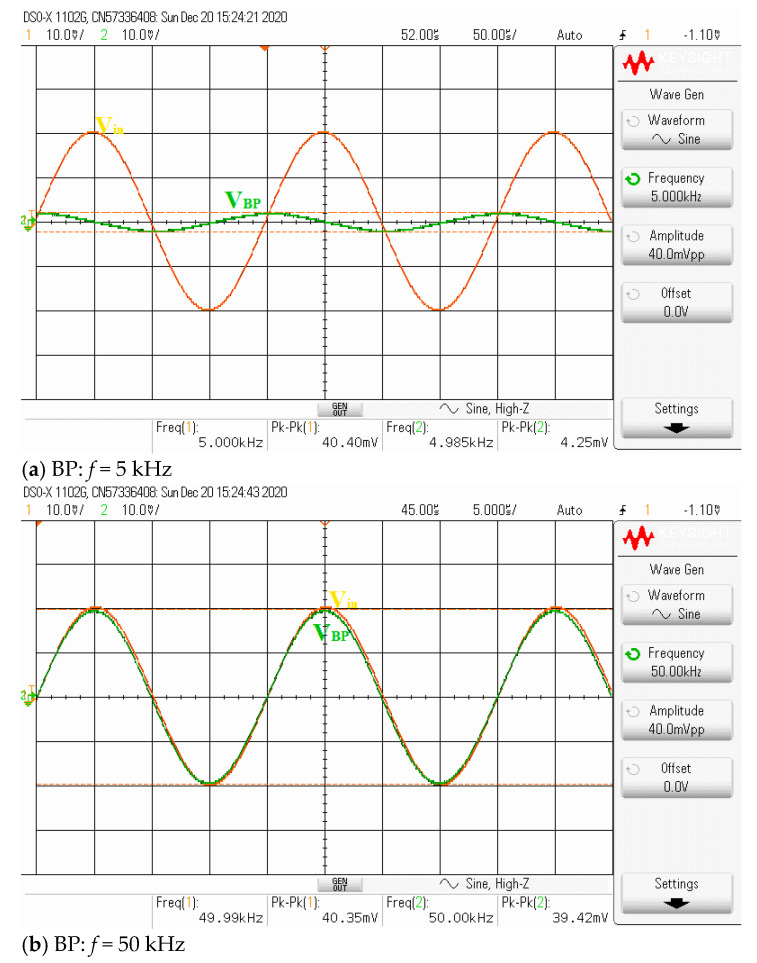

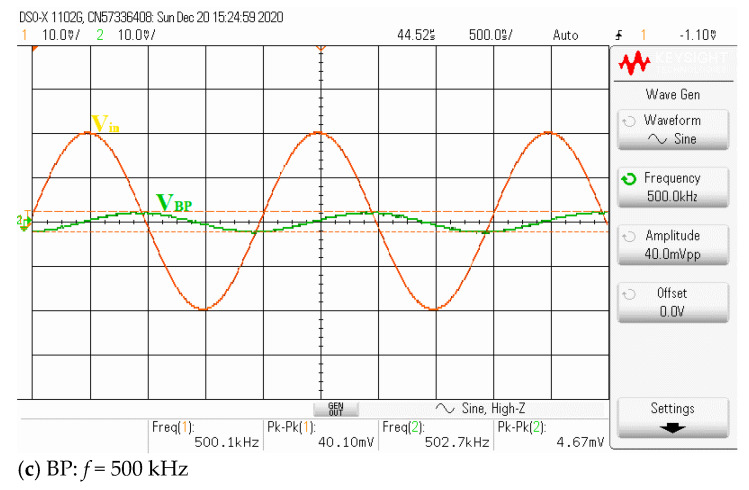
Measured BP filtering transient response.

Theoretically described behaviors of the proposed universal filter were also verified experimentally by implementing the VDDDA from LM13700 (transconductance stage) and AD830 (differential difference amplifier stage), as shown in Figure 19. The transconductance (gm) of LM13700 is electronically controlled with *g_m_* = *I_B_*/2*V_T_*, where *I_B_* is the bias current, and *V_T_* is the thermal voltage. The bias currents and supply voltages were chosen as *I*_*B*1_ = 115 μA, *I*_*B*2_ = 90 μA and *V_CC_* = −*V_EE_* = 5 V, respectively. The values of the grounded capacitors and resistor were chosen as *C*_1_ = *C*_2_ = 5.5 nF and *R*_1_ = 1 kΩ, respectively. The calculated natural frequency and quality factor were *f*_0_ = 50 kHz and *Q* = 1, respectively. Figure 20 shows the frequency characteristics of the LP, HP, BP and BR filters. The experimental natural frequency was *f*_0_ = 49 kHz. However, for the experimental BR response, the attenuation was obtained around −35 dB due to nonideal characteristics of the VDDDA, as explained in Section 2.4. Figure 21 shows the gain response of the band-pass filter at different *I*_*B*2_ values (42 μA, 90 μA and 165 μA). It is observed that the natural frequency is electronically tuned by the bias current *I*_*B*2_ without disturbing the bandwidth or quality factor. The electronic adjustability of the natural frequency via the bias current *I*_*B*1_ is shown in Figure 22. The experimental result shows that the natural frequency *f*_0_ = 26 kHz, 38 kHz and 49 kHz was obtained. The measured input and output waveforms of the BP filtering function are, respectively, shown in Figure 23, where a sine wave signal with a peak-to-peak value of 40 mVpp @ 5 kHz, 50 kHz and 500 kHz was applied to the input of the filter.

**Figure 24:**
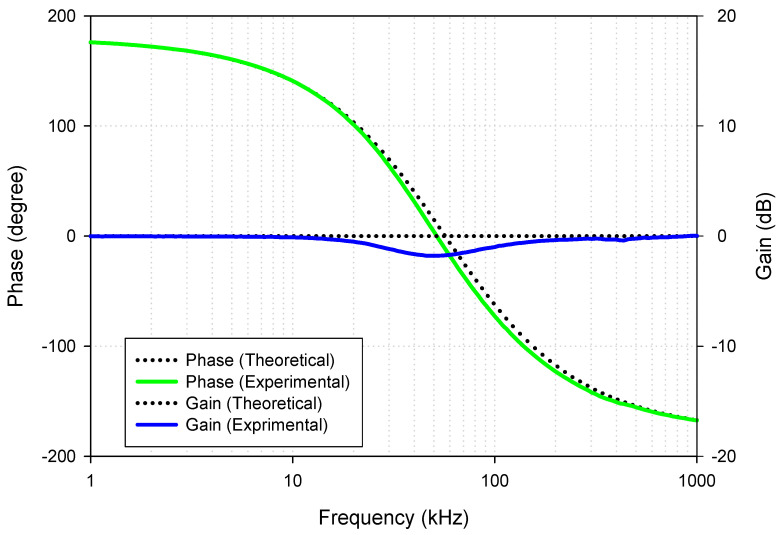
Experimental gain and phase response of the AP filer (*I*_*B*1_ = *I*_*B*2_ = 200μA, *R*_1_ = 1 k and *C*_1_ = *C*_2_ = 5.5 nF).

**Figure 25:**
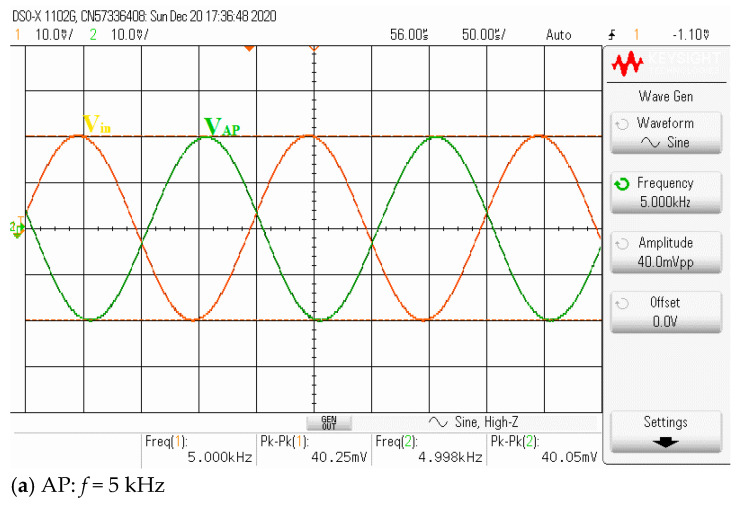

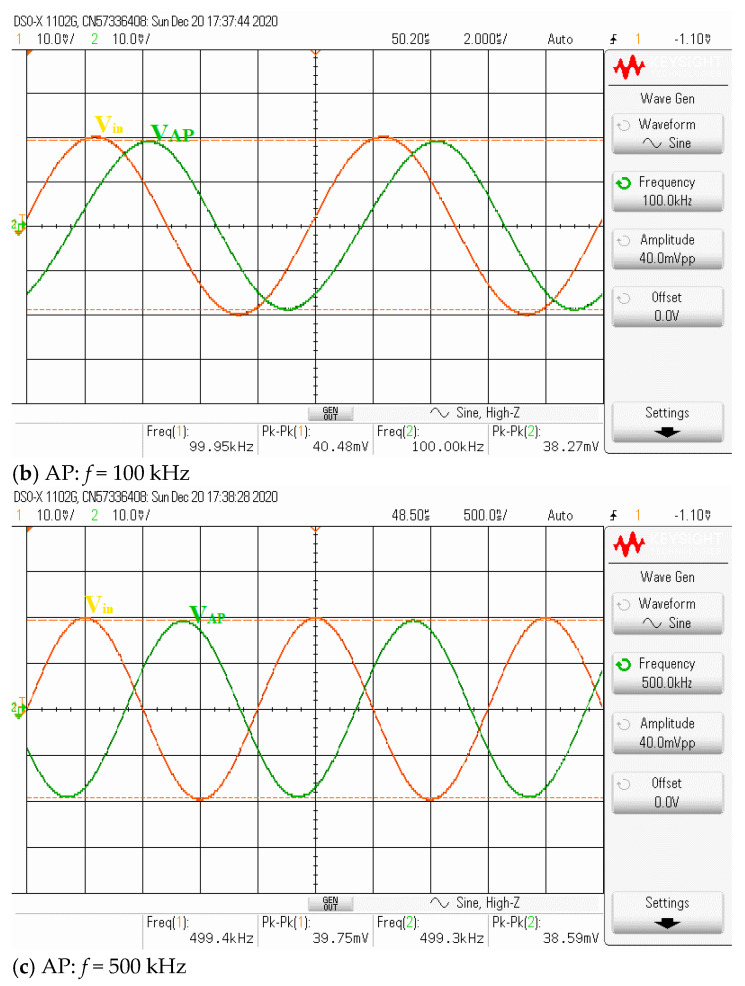
Measured AP filtering transient response.

Next, the inverting all-pass biquad filter with unity voltage gain was tested by setting the transconductances *g*_*m*1_ = *g*_*m*2_ (*I*_*B*1_ = *I*_*B*2_ = 200 μA) and applying the input voltage signal to the input voltage nodes, *v*_*i*1_, *v*_*i*3_ and *v*_*i*4_, while the input voltage node *v*_*i*2_ was grounded. Although it requires the matching conditions of *I*_*B*1_ and *I*_*B*2_, this is the active matching condition that is easier to control than the passive matching condition. The passive elements were still chosen as the resistor *R*_1_ = 1 k and the capacitors *C*_1_ = *C*_2_ = 5.5 nF. Figure 24 shows the experimental phase and magnitude response of the proposed voltage-mode universal biquad filter. The graph shows that the output phase response of the AP filter is shifted from −180 to 180 degrees. However, at the frequency close to the natural frequency, the experimental voltage gain is slightly different from the ideal due to nonideal characteristics of the VDDDA, as explained in Section 2.4. The measured input and output waveforms of the AP filtering function are, respectively, shown in Figure 25a–c, where a sine wave signal with a peak-to-peak value of 40 mVpp @ 5 kHz, 100 kHz and 500 kHz was applied to the input of the filter. It appeared that the proposed filter can be used as the phase shifter circuit with constant output amplitude.

## 5. Comparison

Table 1 compares the proposed universal biquad filter with previous biquad filters using the VDDDA [13,14,15,16,17,18,19,20,21,22,23,24]. These biquad filters are the multiple input multiple output (MIMO) [13,14,23], multiple input single output (MISO) [16,18,19,21,24] and single input multiple output (SIMO) [14,17,20,22] configurations. The design technique used in [13,14,15,17,19,20,21,22] were based on two integrator loops, while the filters in [16,18,23,24] were based on a passive RLC circuit. The proposed filter was designed from the parallel RLC circuit connecting with the voltage differencing circuit, and the VDDDA is very useful for this design. The RLC-based biquad filters proposed in references [16,18,23,24] were very simple circuitry with a single VDDDA as the active building block. Additionally, in [18,24], the *f*_0_ and *Q* were orthogonally controlled via the passive resistor. However, these biquad filters realized from the RLC circuit had the following disadvantages: for example, they consisted of a floating capacitor [16,18,24], the *f*_0_ and *Q* were not orthogonally controlled by changing the separated transconductance of the VDDDA [16,18,23,24], none of the high-impedance nodes and low-impedance nodes were given in references [16,18,23,24] and the unity gain inverting voltage amplifier or double-gain voltage amplifier was required for obtaining several filtering functions [16,18,24]. These limitations of the RLC-based filers have been improved in this work. The two-integrator loop-based biquad filters with high input impedance were obtained from references [13,14,15,17,22,23,24,25], and the low output impedance property for all output nodes was obtained from references [14,22,25]. In [13,14,20,21,22], the *f*_0_ and *Q* were orthogonally controlled by the separated transconductance. Additionally, the two VDDDA-based filters in reference [15] achieved orthogonal control of the *f*_0_ and *Q* via the passive resistor. Additionally, the *f*_0_ and *Q* of the proposed filters in references [13,14,20,21,22] could be linearly and independently controlled by simultaneously setting the transconductances in the integrator circuits. However, the biquad filter proposed in references [13,14,20,21,22] required three VDDDAs. Additionally, the passband voltage gain of the three VDDDA-based filters in references [13,14,22] were not constant during tuning the *f*_0_ and *Q* for some filtering responses. The two-integrator loop filter in references [15,17] could not provide five filter responses. Additionally, two VDDDA-based biquad filters in references [15,17,22] could not achieve orthogonal control of the *f*_0_ and *Q* by separated transconductance. The performances of the universal filers in references [13,14,15,16,17,18,23,24] were proved via simulation only, while both simulation and experiment were verified in this work.

## 6. Conclusions

In this work, a new universal biquad filter using VDDDAs was proposed. The design technique used in this work was based on the parallel RLC circuit connecting with a voltage differencing circuit. The proposed filter is composed of two VDDDAs, one resistor and two grounded capacitors, which ensure reduction in a fabrication space and the compensation of parasitic effects. Five filtering responses with unity passband voltage gain are obtained. The AP response is achieved without matching by connecting the z terminal to the *p* terminal of VDDDA_2_. The *f*_0_ and *Q* are orthogonally controlled from separated transconductance (*f*_0_ is tuned by *g*_*m*1_, and *Q* is tuned by *g*_*m*2_). This feature can be electronically controlled by implementing the VDDDA from the commercially available ICs. The performance and functionality of the proposed universal biquad filter were demonstrated with a simulation and experimental results, confirming the theory.

## Figures and Tables

**Table 1:** Comparison of the proposed biquad filter and other filters using VDDDA.

Ref	Filtering Category	No. of VDDDA	No. of R + C	Use of all Grounded Capacitors	High Impedance of all Input Nodes	Low output Impedance for all Output Node	Electronic Tune of Q without Affecting *ω*_0_	Filtering Functions	Constant Passband Gain during Tuning ω_0_ and *Q* for all Responses	Technology	Additional Circuit	Results	Power supply Voltages & Power Consumption*	Dynamic Range & Noise
[13]	MIMO	3	1 + 2	Yes	Yes	No	Yes	LP, BP, HP, BR, AP	No	0.18 μm TSMC CMOS	No	Simulation	±0.9 V & N/A	N/A
[14]	MIMO	3	1 + 2	Yes	Yes	No	Yes	LP, BP, HP, BR, AP	No	0.18 μm TSMC CMOS	No	Simulation	±0.9 V & N/A	N/A
[15]	SIMO	2	2 + 2	Yes	Yes	No	No	LP, BP, HP	Yes	0.18 μm TSMC CMOS	No	Simulation	±0.9 V & N/A	N/A
[16]	MISO	1	1 + 2	No	No	No	No	LP, BP, HP, BR, AP	Yes	0.25 μm TSMC CMOS	Inverting Amp. & double gain Amp.	Simulation	±1.25 V & 1.58 mW	N/A
[17]	SIMO	2	0 + 2	Yes	Yes	No	No	LP, BP, HP, BR	Yes	0.18 μm TSMC CMOS	No	Simulation	±0.9 V & 0.21 mW	N/A
[18]	MISO	1	2 + 2	No	No	No	No	LP, BP, HP, BR, AP	Yes	0.25 μm TSMC CMOS	Inverting Amp.	Simulation	±1.25 V & N/A	N/A
[19]	MISO	2	0 + 2	Yes	Yes	Yes	No	LP, BP, HP, BR, AP	Yes	Commercial ICs	No	Simulation & Experiment	± 5 V	N/A
[20]	SIMO	3	1 + 2	Yes	Yes	No	Yes	LP, BP, HP, BR, AP	Yes	0.18 μm TSMC CMOS & Commercial ICs	No	Simulation & Experiment	±0.9 V & 0.34 mW	N/A
[21]	MISO	3	1 + 2	Yes	Yes	Yes	Yes	LP, BP, HP, BR, AP	Yes	Commercial ICs	No	Simulation &Experiment	± 5 V & N/A	N/A
[22]	SIMO	3	1 + 2	Yes	Yes	No	Yes	LP, BP, HP, BR, AP	No	0.18 μm TSMC CMOS & Commercial ICs	No	Simulation & Experiment	±0.9 V & N/A	N/A
[23]	MIMO	1	1 + 2	Yes	Yes	No	No	LP, BP	Yes	0.18 μm TSMC CMOS	No	Simulation	±0.9 V & 0.73 mW	N/A
[24]	MISO	1	2 + 2	No	No	No	No	LP, BP, HP, BR, AP	Yes	Commercial ICs	Inverting Amp.	Simulation	±5 V & N/A	N/A
Proposed Filter	MISO	2	1 + 2	Yes	Yes	Yes	Yes	LP, BP, HP, BR, AP	Yes	0.18 μm TSMC CMOS & Commercial ICs	No	Simulation & Experiment	±0.9 V & 0.99 mW	73.27 dB & 46 µVrms

* The power consumption, dynamic range and noise are taken from the simulation. N/A: information not available/shown.
